# Microbiological characteristics of bacteremias among COVID-19 hospitalized patients in a tertiary referral hospital in Northern Greece during the second epidemic wave

**DOI:** 10.1093/femsmc/xtab021

**Published:** 2021-12-02

**Authors:** Efthymia Protonotariou, Paraskevi Mantzana, Georgios Meletis, Areti Tychala, Angeliki Kassomenaki, Olga Vasilaki, Georgia Kagkalou, Ioanna Gkeka, Maria Archonti, Styliani Kati, Simeon Metallidis, Lemonia Skoura

**Affiliations:** Department of Microbiology, AHEPA University Hospital, Thessaloniki 54636, Greece; Medical School, Aristotle University of Thessaloniki, Thessaloniki 54636, Greece; Department of Microbiology, AHEPA University Hospital, Thessaloniki 54636, Greece; Department of Microbiology, AHEPA University Hospital, Thessaloniki 54636, Greece; Medical School, Aristotle University of Thessaloniki, Thessaloniki 54636, Greece; Department of Microbiology, AHEPA University Hospital, Thessaloniki 54636, Greece; Department of Microbiology, AHEPA University Hospital, Thessaloniki 54636, Greece; Department of Microbiology, AHEPA University Hospital, Thessaloniki 54636, Greece; Department of Microbiology, AHEPA University Hospital, Thessaloniki 54636, Greece; Department of Microbiology, AHEPA University Hospital, Thessaloniki 54636, Greece; Department of Microbiology, AHEPA University Hospital, Thessaloniki 54636, Greece; Medical School, Aristotle University of Thessaloniki, Thessaloniki 54636, Greece; Medical School, Aristotle University of Thessaloniki, Thessaloniki 54636, Greece; First Department of Internal Medicine, AHEPA University Hospital, Thessaloniki 54636, Greece; Department of Microbiology, AHEPA University Hospital, Thessaloniki 54636, Greece; Medical School, Aristotle University of Thessaloniki, Thessaloniki 54636, Greece

**Keywords:** COVID-19, bacteremia, coinfection

## Abstract

Northern Greece was struck by an intense second COVID-19 (coronavirus disease 2019) epidemic wave during the fall of 2020. Because of the coinciding silent epidemic of multidrug-resistant organisms, the handling of COVID-19 patients became even more challenging. In the present study, the microbiological characteristics of bacteremias in confirmed cases of hospitalized COVID-19 patients were determined. Data from 1165 patients hospitalized between September and December 2020 were reviewed regarding the frequency of bloodstream infections, the epidemiology and the antibiotic susceptibility profiles of the causative bacteria. The hospital's antibiotic susceptibility data for all major nosocomial pathogens isolated from bacteremias of COVID-19 patients between September and December 2020 versus those between September and December 2019 were also compared. Overall, 122 patients developed bacteremia (10.47%). The average of time interval between hospitalization date and development of bacteremia was 13.98 days. Admission to ICU occurred in 98 out of 122 patients with an average stay time of 15.85 days and 90.81% in-hospital mortality. In total, 166 pathogens were recovered including 114 Gram-negative bacteria and 52 Gram-positive cocci. *Acinetobacter baumannii* was the most frequent (*n* = 51) followed by *Klebsiella pneumoniae* (*n* = 45) and *Enterococcus faecium* (*n* = 31). Bacteremias in hospitalized COVID-19 patients were related with prolonged time of hospitalization and higher in-hospital mortality, and the isolated microorganisms represented the bacterial species that were present in our hospital before the COVID-19 pandemic. Worryingly, the antibiotic resistance rates were increased compared with the pre-pandemic era for all major opportunistic bacterial pathogens. The pandemic highlighted the need for continuous surveillance of patients with prolonged hospitalization.

## Teaser

COVID-19 patient bacteremias in a Greek referral hospital.

## INTRODUCTION

Since its emergence in December 2019 (Ye *et al*. 2020), severe acute respiratory syndrome coronavirus-2 (SARS-CoV-2) has caused a pandemic accounting for >228 394 572 cases and 4 690 186 deaths worldwide as of 20 September 2021 (https://covid19.who.int/). As it has already been observed in previous epidemics due to respiratory viruses, bacterial secondary infections are frequent among hospitalized patients and attributed to worsening outcomes regarding patient's morbidity and mortality (MacIntyre *et al*. 2018).

Secondary bacterial infections in coronavirus disease 2019 (COVID-19) are associated with prolonged ICU stay and mechanical ventilation, higher occurrence of ARDS and higher mortality (Bardi *et al*. 2021; De Santis *et al*. 2021). Among them, bloodstream infections (BSIs) play a significant role with a prevalence varying from 5.5% to 40% in COVID-19 patients across different settings (Bardi *et al*. 2021; Bhargava *et al*. 2021; De Santis *et al*. 2021; Kokkoris *et al*. 2021; Palanisamy *et al*. 2021).

The source of bacteremias is mainly attributed to pulmonary infections (Gomez-Simmonds *et al*. 2021; Bhargava *et al*. 2021). Moreover, factors like the presence of central venous catheter, prolonged ICU stay, the use of immunosuppressants in COVID-19 treatment, such as corticosteroids, anakinra and tocilizumab, and the gut microbiome dysbiosis further contribute to progression to bacteremia among COVID-19 patients (De Santis *et al*. 2021; Khatri *et al*. 2021; Nori *et al*. 2021; Venzon *et al*. 2021).

The prevalence of pathogens causing bacteremia is variable across different settings from around the world. Reports from the United States show that Gram-positive bacteria prevail over Gram-negative bacteria with *Staphylococcus aureus* being predominant (Bhargava *et al*. 2021; Nori *et al*. 2021). Likewise, in a single-center study in northern Italy, coagulase-negative staphylococci, *Enterococcus faecalis* and *S. aureus* were the most frequently isolated bacteria from blood cultures of ICU patients (Giacobbe *et al*. 2020). On the other hand, in India and Greece among bacteremias in COVID-19 ICU patients, multidrug-resistant (MDR) Gram-negative bacteria are the more common isolates with *Acinetobacter baumannii* being predominant (Kokkoris *et al*. 2021; Palanisamy *et al*. 2021).

Additionally, there are reports outlining a rise in MDR pathogens during the pandemic. In particular, Gomez-Simmonds *et al*. report an increase in the detection of carbapenemase-producing Enterobacterales among COVID-19 patients compared with the rates observed in the previous years at a medical center in New York City (Gomez-Simmonds *et al*. 2021), whereas in another study from Turkey, the researchers observed an increase in *A. baumannii* infections accompanied by a decrease in ESBL (extended-spectrum beta-lactamase)-producing Enterobacterales (Karataş *et al*. 2021).

The present study aims to assess the prevalence, frequency and distribution of microorganisms as well as their antimicrobial susceptibility in confirmed cases of COVID-19 patients with bacteremia hospitalized in our institution.

## MATERIALS AND METHODS

We conducted a retrospective observational study of COVID-19 patients admitted between 1 September 2020 and 31 December 2020 in AHEPA University Hospital, a 700-bed tertiary care hospital in Thessaloniki, Greece. AHEPA hospital serves as one of the reference hospitals for COVID-19 patients in Northern Greece. The diagnosis of COVID-19 was performed by real-time PCR, using either the Abbott Molecular RealTime or the NeuMoDx SARS-CoV-2 assay. Blood cultures were performed upon clinical suspicion of bacteremia. All patients with a positive SARS-CoV-2 PCR test result who developed BSI were included. The frequency and distribution of BSIs, epidemiology and antibiotic profiles of the causative bacteria were analyzed.

Blood cultures positive for fungi and those with positive skin flora that did not grow in multiple cultures were excluded. Blood culture vials were incubated in the BACTEC FX instrument (Becton Dickinson, Franklin Lakes, NJ) for a maximum of 5 days. Bacterial identification and antimicrobial susceptibility testing were performed with the VITEK 2 automated system (bioMérieux, France). The results were interpreted using the European Committee on Antimicrobial Susceptibility Testing (EUCAST) v 11.0 breakpoints (https://www.eucast.org/clinical_breakpoints). According to the EUCAST guidelines, minimum inhibitory concentration values for colistin were determined with broth microdilution using the automated system MICRONAUT-S (Merlin, Germany). Multidrug-resistant organism (MDRO) was defined as acquired non-susceptibility to at least one agent in three or more antimicrobial categories (Magiorakos *et al*. 2012). In our study, this category included methicillin-resistant *S. aureus* (MRSA), vancomycin-resistant enterococci (VRE), ESBLs and carbapenem-resistant Gram-negative bacteria such as carbapenem-resistant *A. baumannii* and carbapenem-resistant Enterobacterales (CRE). Among CRE, carbapenemase and ESBL activity was assessed; double-disk synergy test with the addition of EDTA and phenylboronic acid on meropenem disks was used for the phenotypical detection of MBL and KPC producers, respectively, and the modified ESBL test for the detection of ESBLs (Tsakris *et al*. 2010; Poulou *et al*. 2014). In case an MBL was detected, the modified Hodge test was performed in order to differentiate between NDM and VIM carbapenemases (CLSI 2015). Isolates were furtherly tested with the AMR Direct Flow Chip Kit DNA microarray (Master Diagnóstica, Spain) for important carbapenemases, including KPC, GES, VIM, IMP, NDM, OXA-23, OXA-51 and OXA-48 (Protonotariou *et al*. 2021).

The hospital's antimicrobial susceptibility data for all major nosocomial pathogens isolated from bacteremias of COVID-19 patients between September and December 2020 versus those between September and December 2019 were compared. The chi-square or the Fisher's exact test where appropriate was applied to compare data and statistical significance was set to *P* < 0.05. Statistical analyses were performed using SPSS 21.0. No specific approval from our institutional review board was required and no informed consent was needed for this study since data were taken as part of the standard patient care and used anonymously.

## RESULTS

Between September and December 2020, 1165 (690 male and 475 female) patients with confirmed COVID-19 were hospitalized in our institution. Their median age was 64 years. Among them, 122 patients (86 males and 36 females) developed bacteremia (10.47%). The median age of all patients with BSIs was 66 years (Flowchart S1, Supporting Information).

In our study, the blood culture positivity rate for all hospitalized patients increased from 21.6% (387/1793) to 33.4% (495/1481) comparing the data from September to December 2019 with those from September to December 2020, while the respective rates especially for patients admitted to the ICU were 54.3% (50/92) and 60.58% (206/340).

One hundred nineteen out of one hundred twenty-two patients developed secondary bacteremia (>48 h from their hospital admission) (Garcia-Vidal *et al*. 2021). The average time interval between initial hospitalization date and development of bacteremia was 12.8 days. The in-hospital mortality in this group was 84.42% (103/122). Admission to ICU occurred in 98 out of 122 patients with an average length of stay being 15.85 days and 90.81% in-hospital mortality. The number of patients without secondary bacteremia was 981 and their in-hospital mortality was 19.77%. Forty-four out of 981 were admitted to the ICU with an average stay time of 8.07 days and 86.36% in-hospital mortality.

In total, 166 pathogens were recovered from 122 patients—114 Gram-negative bacteria and 52 Gram-positive cocci. The most frequently isolated organisms were *A. baumannii* (*n* = 51), *Klebsiella pneumoniae* (*n* = 45), *Enterococcus faecium* (*n* = 31), *E. faecalis* (*n* = 16) and *Pseudomonas aeruginosa* (*n* = 9) (Table 1).

**Table 1.** Microorganisms recovered by bacteremias among COVID-19 patients.

Microorganism	*n*	MDR Gram-negative bacteria/VRE/MRSA
*Acinetobacter baumannii*complex	51	51
*Klebsiella pneumoniae*	45	43
*Pseudomonas aeruginosa*	9	4
*Acinetobacter junii*	1	0
*Klebsiella oxytoca*	1	0
*Enterobacter cloacae* complex	2	2
*Proteus mirabilis*	1	0
*Citrobacter koseri*	1	0
*Achromobacter xylosoxidans*	1	1
*Raoultella planticola*	1	0
*Sphingomonas paucimobilis*	1	0
*Enterococcus faecium*	31	9
*Enterococcus faecalis*	16	1
*Enterococcus gallinarum*	1	0
*Staphylococcus aureus*	3	2
*Streptococcus mitis*	1	0
**Total**	**166**	**113**

The isolations of *K. pneumoniae*,*A. baumannii* and *E. faecium* increased substantially between September and December 2020 compared with those between September and December 2019 (45 cases vs 15 for *K. pneumoniae*, 51 cases vs 31 for *A. baumannii*and 31 cases vs 12 for*E. faecium*).

MDROs were observed in 101/114 of Gram-negative bacteria and in 12/52 of Gram-positive cocci. The majority of MDROs were *A. baumannii*,*K. pneumoniae* and *Enterococcus* spp. All *A. baumannii* were resistant to carbapenems and presented an extensively drug-resistant profile. Resistance rates of *Acinetobacter* strains to amikacin, gentamicin and trimethoprim–sulfamethoxazole were >90% and 29.41% to colistin. The majority of *K. pneumoniae* strains were MDR exhibiting >95% resistance to ceftazidime, carbapenems and piperacillin–tazobactam; 68.89% to fosfomycin; 46.67% to amikacin; and 35.56% to gentamicin and colistin. A high percentage of *P. aeruginosa* isolates (44.44%) were resistant to ceftazidime, piperacillin–tazobactam, carbapenems and gentamicin; 33.33% to amikacin; and 11.11% to colistin. Nine out of 31 *E. faecium* were resistant to vancomycin (VRE) and two out of three *S. aureus* were MRSA (Table 2).

**Table 2.** Antimicrobial MDR profiles of major nosocomial pathogens isolated from bacteremias of COVID-19 patients during September–December 2020 and from bacteremias during the same period in 2019.

	COVID-19 September–December 2020	September–December 2019		COVID-19 September–December 2020	September–December 2019	
Microorganism	*N*	*N*	Antimicrobial	*R*% (*n*)	*R*% (*n*)	*P*
*Acinetobacter baumannii*complex	51	31	Imipenem	100% (51/51)	100% (31/31)	-
			Meropenem	100% (51/51)	100% (31/31)	-
			Amikacin	94.00% (47/50)	89.66% (26/29)	0.664
			Gentamicin	92.00% (46/50)	75.86% (22/29)	0.088
			Colistin	29.41% (15/51)	6.45% (2/31)	0.013
			Trimethoprim–sulfamethoxazole	90% (45/50)	75.86% (22/29)	0.112
*Klebsiella pneumoniae*	45	15	Ceftazidime	95.56% (43/45)	61.54% (8/13)	0.005
			Piperacillin–tazobactam	97.78% (44/45)	69.23% (9/13)	0.007
			Imipenem	95.56% (43/45)	46.67% (7/15)	<0.005
			Meropenem	95.56% (43/45)	46.67% (7/15)	<0.005
			Amikacin	46.67% (21/45)	38.46% (5/13)	0.600
			Gentamicin	35.56% (16/45)	30.77% (4/13)	1.000
			Fosfomycin	68.89% (31/45)	41.67% (5/12)	1.002
			Colistin	35.56% (16/45)	0% (0/14)	0.007
			Trimethoprim–sulfamethoxazole	40% (16/40)	61.54% (8/13)	0.175
*Pseudomonas aeruginosa*	9	12	Ceftazidime	44.44% (4/9)	50.00% (6/12)	1.000
			Piperacillin–tazobactam	44.44% (4/9)	50.00% (6/12)	1.000
			Imipenem	44.44% (4/9)	50.00% (6/12)	1.000
			Meropenem	44.44% (4/9)	50.00% (6/12)	1.000
			Amikacin	33.33% (3/9)	41.67% (5/12)	1.000
			Gentamicin	44.44% (4/9)	41.67% (5/12)	1.000
			Colistin	11.11% (1/9)	0% (0/12)	0.429
*Enterococcus faecium*	31	12	Vancomycin	29.03% (9/31)	33.33% (4/12)	1.000
*Staphylococcus aureus*	3	21	Cefoxitin screen	66.67% (2/3)	61.90% (13/21)	1.000

The hospital's susceptibility data comparison between September and December 2019 for all hospitalized patients with a variety of morbidities versus those between September and December 2020 COVID-19 patients revealed an increase by >10% for *A. baumannii* in gentamicin and trimethoprim–sulfamethoxazole resistance, and as for *K. pneumoniae* a rise in resistance to ceftazidime (34%), piperacillin–tazobactam (28.55%) and fosfomycin (27.2%). A remarkable increase of *K. pneumoniae* carbapenem resistance rates (almost 50%) was observed between 2019 and 2020. Resistance to colistin increased by >10% for *A. baumannii* (22.96%), *K. pneumoniae* (35.56%) and *P. aeruginosa* (11.11%) (Table 2).

Phenotypic detection revealed carbapenemase production in all carbapenem-resistant Enterobacterales, while ESBL production was observed among 20 *K. pneumoniae* and all two *Enterobacter cloacae* isolates. Molecular investigation of carbapenemase production among carbapenem-resistant isolates revealed that *K. pneumoniae* harbored KPC (34/43) and NDM (9/43), *E. cloacae* VIM (2/2), *P. aeruginosa* VIM (2/4), and *A. baumannii* OXA-23 and OXA-51 carbapenemases.

## DISCUSSION

Bacterial coinfections (acute bacterial infection presented with SARS-CoV-2 infection simultaneously) and secondary infections (emerging during the course of illness or hospital stay) in COVID-19 patients became evident early in the pandemic (Zamora-Cintas *et al*. 2021). Even though the rate of secondary infections may vary among hospitals and is poorly defined globally (Lardaro *et al*. 2021), high occurrence with significant impact on prognosis (Suarez-de-la-Rica *et al*. 2021) and high mortality rates have been documented (Nori *et al*. 2021). BSIs seem to be very frequent among mechanically ventilated COVID-19 patients (Risa *et al*. 2021) together with upper respiratory tract bacterial and fungal superinfections (Mazzariol *et al*. 2021). A recent multicenter study from Italy showed that BSIs were a common secondary infection and were more frequent during the pandemic than in the same pre-COVID-19 time period (Pasquini *et al*. 2021).

In 2020, we observed a notable rise in BSIs accompanied by more resistant phenotypes of the isolated bacteria when compared with the respective rates of the previous year. Notably, this variability of the resistance rates could not be explained as part of an expected year-to-year variability happening even before the pandemic. Moreover, the incidence of BSIs in COVID-19 patients in our hospital was one of the highest published in the literature, whereas the more prevalent causative agents among them were Gram-negative bacteria, the majority of which were MDROs.

In our study, BSIs occurred in 122/1165 (10.47%) COVID-19 patients, which is in agreement with recent studies where the occurrence varied from 8.6% to 9.05% (Bhargawa *et al*. 2021; Pasquini *et al*. 2021), showing that BSIs are common complications in this category of patients and the occurrence is higher than the reported 3–3.5% in early studies (Sepulveda *et al*. 2020). The microorganisms responsible for secondary infections in hospitalized COVID-19 patients may vary (Hughes *et al*. 2020; Bhargawa *et al*. 2021; Kokkoris *et al*. 2021; Mazzariol *et al*. 2021; Risa *et al*. 2021) and it is logical to presume that the bacterial hospital epidemiology of each hospital plays an important role. Our study showed a substantial rise of hospital onset of infections caused by members of the ESKAPE (*E. faecium*,*S. aureus*,*K. pneumoniae*,*A. baumannii*,*P. aeruginosa* and *Enterobacter* spp.) group of bacteria, which are well known for their virulent as well as MDR profile (Mulani *et al*. 2019). The rise was observed especially among *E. faecium*,*K. pneumoniae* and *A. baumannii* pathogens both in number of isolations and in their MDR profile compared with previous years. Similarly, the types of carbapenemase-encoding genes among CRE and *A. baumannii* were in accordance with those that have been circulating in our hospital setting before the pandemic. This observation could be possibly explained by the increased number of COVID-19 patient admissions during the surge, their prolonged time of hospitalization and the extensive antimicrobial treatment that these patients received. Moreover, the personnel dedicated to infection control were constrained.

Our study has several limitations. First of all, it is a single-center study and the implicated bacteria might represent a site-specific pathogen profile. Second limitation is the lack of detailed clinical data regarding the source of infection, type of antibiotic treatment and immunomodulators. No genomic analysis for epidemiological association was performed, as this was beyond the scope of our study.

Despite these limitations, our study is the first to our knowledge that gives the prevalence and spectrum of secondary bacterial BSIs in Greece including a large number of patients and comparing data with those retrieved at the same period 1 year ago. This way, we aspired to present a notable insight into the impact of the pandemic on BSIs and bacterial resistance rates among hospitalized patients in our region. Overall, our study has revealed high rates of MDR ESKAPE BSIs among the hospitalized COVID-19 patients—a finding with significant implications for active surveillance and reinforcement of hospital infection prevention practices as well as for clinical management with the appropriate antibiotic therapies for secondary infections during the ongoing COVID-19 pandemic.

## Supplementary Material

xtab021_Supplemental_File
