# Supplementary figures and images for: Microbiological characteristics of bacteremias among COVID-19 hospitalized patients in a tertiary referral hospital in Northern Greece during the second epidemic wave
Source: FEMS Microbes. 2021 Dec 2;2:xtab021. doi: 10.1093/femsmc/xtab021 (PMC8847882; doi:10.1093/femsmc/xtab021)

## Flowchart

Study period: Sept 1 – Dec 31, 2020

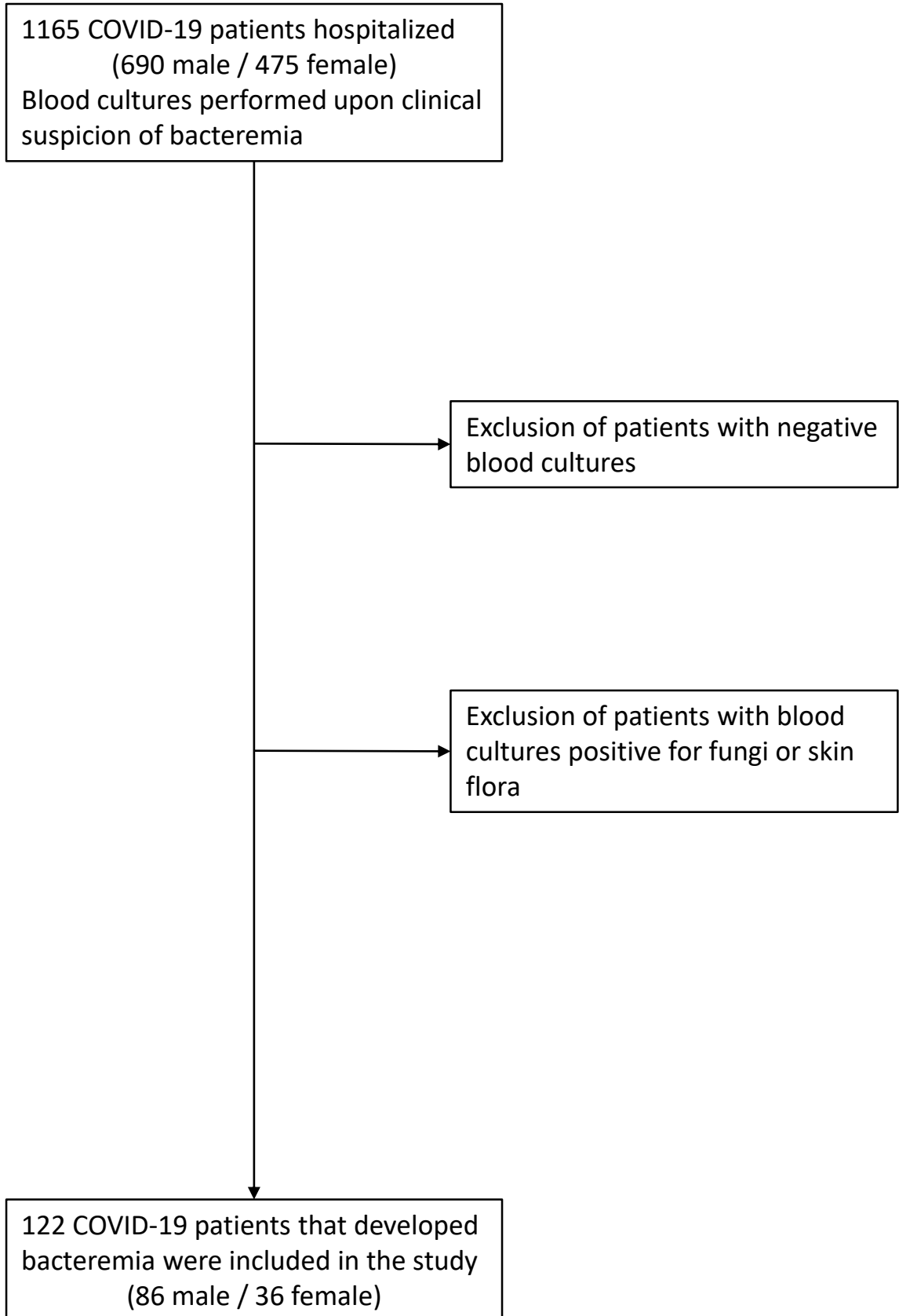

Supplement: xtab021_Supplemental_File [file xtab021_supplemental_file.pdf]
